# Quick and Spontaneous Transformation between [3Fe–4S] and [4Fe–4S] Iron–Sulfur Clusters in the tRNA-Thiolation Enzyme TtuA

**DOI:** 10.3390/ijms24010833

**Published:** 2023-01-03

**Authors:** Masato Ishizaka, Minghao Chen, Shun Narai, Yoshikazu Tanaka, Toyoyuki Ose, Masaki Horitani, Min Yao

**Affiliations:** 1 Graduate School of Life Science, Hokkaido University, Kita 8, Nishi 5, Kita-ku, Sapporo 060-0810, Japan; 2 Faculty of Advanced Life Science, Hokkaido University, Kita 8, Nishi 5, Kita-ku, Sapporo 060-0810, Japan; 3 Graduate School of Life Sciences, Tohoku University, 2-1-1 Katahara, Aoba-ku, Sendai 980-8577, Japan; 4 Faculty of Agriculture, Department of Applied Biochemistry and Food Science, Saga University, 1 Honjo-machi, Saga 840-8502, Japan; 5 The United Graduate School of Agricultural Science, Kagoshima University, 1-21-24 Korimoto, Kagoshima 890-0065, Japan

**Keywords:** iron–sulfur cluster, tRNA-thiolation, EPR spectroscopy, sulfurtransferase, tRNA sulfur modification, 2-thiouridine biosynthesis, redox biochemistry

## Abstract

Iron–sulfur (Fe–S) clusters are essential cofactors for enzyme activity. These Fe–S clusters are present in structurally diverse forms, including [4Fe–4S] and [3Fe–4S]. Type-identification of the Fe–S cluster is indispensable in understanding the catalytic mechanism of enzymes. However, identifying [4Fe–4S] and [3Fe–4S] clusters in particular is challenging because of their rapid transformation in response to oxidation–reduction events. In this study, we focused on the relationship between the Fe–S cluster type and the catalytic activity of a tRNA-thiolation enzyme (TtuA). We reconstituted [4Fe–4S]-TtuA, prepared [3Fe–4S]-TtuA by oxidizing [4Fe–4S]-TtuA under strictly anaerobic conditions, and then observed changes in the Fe–S clusters in the samples and the enzymatic activity in the time-course experiments. Electron paramagnetic resonance analysis revealed that [3Fe–4S]-TtuA spontaneously transforms into [4Fe–4S]-TtuA in minutes to one hour without an additional free Fe source in the solution. Although the TtuA immediately after oxidation of [4Fe–4S]-TtuA was inactive [3Fe–4S]-TtuA, its activity recovered to a significant level compared to [4Fe–4S]-TtuA after one hour, corresponding to an increase of [4Fe–4S]-TtuA in the solution. Our findings reveal that [3Fe–4S]-TtuA is highly inactive and unstable. Moreover, time-course analysis of structural changes and activity under strictly anaerobic conditions further unraveled the Fe–S cluster type used by the tRNA-thiolation enzyme.

## 1. Introduction

Iron–sulfur (Fe–S) clusters, important cofactors of proteins (Fe–S proteins), are responsible for electron transition, protein stabilization, ligand binding, etc. [1]. The Fe–S clusters are essential for various cellular processes such as photosynthesis, respiration, nitrogen fixation, and oxygen sensing [2,3,4,5]. Recently, Fe–S clusters were found to regulate DNA replication, DNA repair [6,7], RNA replication, and RNA regulation [8,9]. These findings suggested that the biological roles of Fe–S clusters are more extensive than speculated previously. Fe–S clusters differ structurally in terms of a varying number of Fe and S atoms, for example, [2Fe–2S], [3Fe–4S], [4Fe–3S], [4Fe–4S], [4Fe–5S], [8Fe–7S], and [8Fe–9S] [10]. Identification of the type of Fe–S cluster is essential to understand the catalytic mechanism of Fe–S protein. However, accurate structural determination of [4Fe–4S] and [3Fe–4S] is challenging because the structural difference involves only one Fe atom (hereafter termed as the unique Fe), and [4Fe–4S] is sensitive to oxidation and readily decays to [3Fe–4S] [11,12]. [4Fe–4S] and [3Fe–4S] are often been misidentified in enzymes, such as aconitase [13], pyruvate formate-lyase activating enzyme [14], and isopentenyl-diphosphate [15]. Although previous studies have shown structural changes as a cause of misidentification between [4Fe–4S] and [3Fe–4S] [11,16,17], reports on the time-course-dependent structural changes under strictly anaerobic control are lacking to date. 

Recently, the identification of [3Fe–4S] and [4Fe–4S] in tRNA-thiolation (sulfur modification) enzymes has raised the attention of researchers widely. These enzymes catalyze the replacement of oxygen with sulfur on tRNA [18], which improves the translational fidelity, thermal stability, and UV sensing of tRNA, depending on the position of the substrate base [19,20,21]. Loss of thiolation at the wobble position of tRNA (anticodon 1 position) correlates with the development of mitochondrial disease myoclonus epilepsy with ragged-red fibers, and excess thiolation promotes cancer and metastasis [22,23]. Studies have identified several tRNA-thiolation enzymes, such as TtuA [24,25,26], TtcA [27], and Ncs6 [28,29] of the TtuA/Ncs6 family; MnmA [30,31] (MnmA family); and ThiI [29] (ThiI family) (Tables S1 and S2). TtuA/Ncs6 family enzymes share a PP-loop (SGGXD [S/T]) residue-motif in the pyrophosphatase (PPase) domain and a CXXC…C residue-motif for ATP and Fe–S cluster binding, respectively [28]. In contrast, some MnmA proteins use the DXXC…C motif, and some ThiI proteins do not possess Fe–S clusters. The PP-loop is a typical ATP binding motif involved in ATP hydrolysis, and CXXC…C motif is imperative for coordinating the Fe–S cluster [28]. However, the reported characterization of Fe–S clusters in these enzymes is controversial. For example, it was recently reported that bacterial MnmA enzyme possesses a [4Fe–4S] cluster [30,31], which is contrary to a previous report depicting that *Eco*MnmA is independent of the Fe–S cluster [32]. Furthermore, the spectroscopic and biochemical analysis revealed that Ncs6 catalyzes tRNA-thiolation using [3Fe–4S] [29]; however, the structure of [4Fe–4S]-Ncs6 was determined under anaerobic conditions [28]. Previously, we have reported that the non-cysteine chelated Fe (unique Fe) in [4Fe–4S]-TtuA coordinates with the C-terminus of the sulfur donor protein TtuB (TtuB–COOH) [24,25], suggesting the importance of unique Fe in transferring sulfur from TtuB–COSH to tRNA. Considering the high sequence identity between tRNA-thiolation enzymes (Figure S1, Table S2) and the similarity of tRNA-thiolation reactions (Figure S2), the findings for Ncs6 raise the question of whether TtuA only requires [4Fe–4S] or both [4Fe–4S] and [3Fe–4S] clusters to perform its function. 

In this study, we reconstituted [4Fe–4S]-TtuA and oxidized it to [3Fe–4S]-TtuA under strictly anaerobic conditions. Then we analyzed the structure of the Fe–S cluster using electron paramagnetic resonance (EPR) in time-course experiments and evaluated the activity of these samples. Results from EPR spectroscopy revealed that the [3Fe–4S] cluster in TtuA spontaneously transformed into [4Fe–4S] with the unique Fe in minutes to one hour without an additional free Fe source in the solution. The tRNA-thiolation assay of these samples revealed that while TtuA was inactive immediately after the oxidation of [4Fe–4S]-TtuA to [3Fe–4S]-TtuA, its activity gradually recovered to a level comparable with [4Fe–4S]-TtuA, which was corresponding to an increase in [4Fe–4S]-TtuA. Furthermore, we found that [3Fe–4S]-TtuA could not interact with the sulfur donor at the C-terminus of TtuB, indicating that TtuA requires [4Fe–4S] only for 5-methyl-2-thiouridine (m^5^s^2^U) biosynthesis (tRNA-thiolation) at position 54 of tRNA. Considering the sequence similarity, especially the highly conserved active residues in the TtuA/Ncs6 family, we propose that the unique Fe of [4Fe–4S] is essential for TtuA/Ncs6 family members to catalyze tRNA-thiolation. Our findings reveal that the correlation analysis of time-course between the structural changes of cofactor Fe–S clusters and the enzymatic activity of that protein under strictly anaerobic conditions is necessary to reveal the reaction mechanism. 

## 2. Results

### 2.1. [3Fe–4S] Cluster in TtuA was Spontaneously Transformed into the [4Fe–4S] Cluster

Since [4Fe–4S]^2+^ cluster and Fe^2+^ are EPR-silent, we evaluated [4Fe–4S]^1+^, [3Fe–4S]^1+^ clusters, and free Fe^3+^ ions using EPR spectroscopy to monitor the changes in Fe–S cluster bound to TtuA under strictly anaerobic conditions. We transformed reconstituted [4Fe–4S]^2+^-TtuA into [4Fe–4S]^1+^–TtuA by adding the reductant dithionite (DT) to obtain the spectra of [4Fe–4S]-TtuA [25] and evaluated [3Fe–4S]^1+^ cluster and free Fe^3+^ ions for oxidized [4Fe–4S]-TtuA (Figure S3). 

First, we confirmed whether excess DT affects the structures of Fe–S clusters in TtuA. The linear shape of EPR spectra and the *g*-value of [4Fe–4S]^1+^–TtuA did not alter until 30 min after reduction, showing that reduction with DT does not cause structural changes in the Fe–S cluster bound to TtuA (Figure S4). 

**Figure 1:**
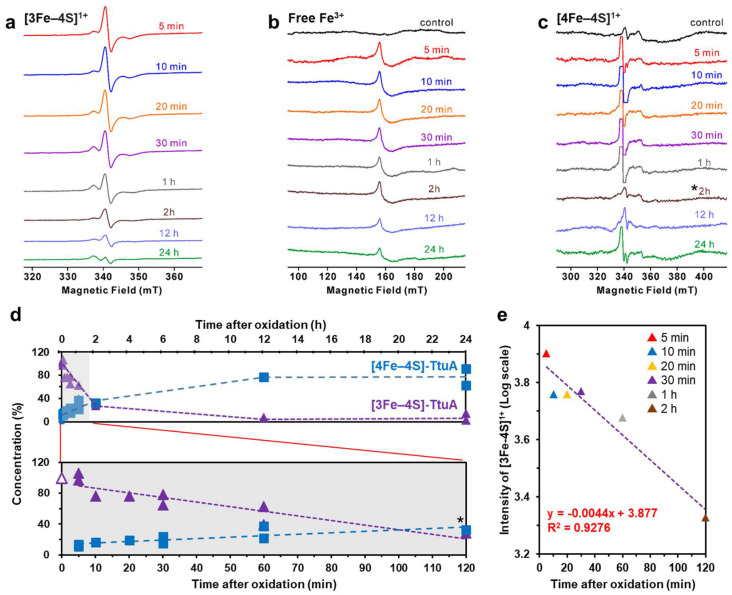
Structural changes in the Fe–S cluster bound to TtuA. [3Fe–4S]^1+^ (**a**), free Fe^3+^ (**b**), and [4Fe–4S]^1+^ (**c**) EPR spectra of K_3_[Fe(CN)_6_]-oxidized TtuA were detectable in time-course. (**d**) Top: The plot of structural changes in Fe–S clusters in time-course, which was estimated from the EPR spectra of (**a**,**c**). Bottom: close-up view of 0–120 min in (**d**) (top). We conducted EPR experiments twice at 5, 30, 60 min, and 24 h. Note that the amount of [3Fe–4S] immediately after oxidation (0 min) was estimated as 100% (indicated in a purple triangle) from the calibration curve because measurement at 0 min is impossible. (**e**) Calibration curve of the signal intensity of [3Fe–4S]^1+^ versus time after oxidation. The color code of each point is the same as that in (**a**). Asterisks (*) in (**c**,**d**) indicate a weak signal with a relatively larger experiment error.

Next, for preparing [3Fe–4S]-TtuA, we oxidized [4Fe–4S]-TtuA by adding ferricyanide (K_3_[Fe(CN)_6_]), and then removed excess K_3_[Fe(CN)_6_] and free Fe from the solution with the desalting column. The EPR spectra of freshly oxidized TtuA showed that the [3Fe–4S]^1+^ cluster was bound to TtuA (g = [2.02, 2.01, 1.97] at 40 K) (Figure 1a) [25]. Moreover, we also observed a peak of free Fe^3+^ (g = 4.3 at 12 K) (Figure 1b) [33]. Considering that free Fe and excess K_3_[Fe(CN)_6_] in the solution were removed through desalination, free Fe should be derived from the degradation of [3Fe–4S] from [3Fe–4S]-TtuA. After 5, 10, 20, and 30 min and 1, 2, 12, and 24 h of desalination under strictly anaerobic conditions, signal intensity peaks of [3Fe–4S]^1+^ cluster and free Fe in the samples decreased with time (Figure 1a,b). The EPR spectra of [3Fe–4S]-TtuA, without desalination (containing free Fe and excess K_3_[Fe(CN)_6_]), showed a decrease in [3Fe–4S] cluster similar to that after removing excess K_3_[Fe(CN)_6_] and free Fe (Figure S5). In addition, to evaluate the presence of the [4Fe–4S] cluster in all test samples, we reduced the samples with DT and recorded their EPR spectra. The signal intensity of the [4Fe–4S]^1+^ cluster increased with time as the decrease in the signal intensity of the [3Fe–4S] cluster (Figure 1c,d).

Since quantifying the amount of [3Fe–4S] cluster immediately after oxidation (0 min) is challenging due to experimental limitations, we estimated an approximately linear correlation coefficient of 3.877 for the signal intensity of [3Fe–4S] as a standard value to calculate the amount of [3Fe–4S] cluster (Figure 1e). When we normalized the amount of [4Fe–4S] cluster to 100% in TtuA before oxidation, [3Fe–4S]-TtuA was estimated to contain ~10% [4Fe–4S] cluster after 5 min of oxidation. Nearly one-third of [3Fe–4S]-TtuA was transformed to [4Fe–4S]-TtuA after 1 h of oxidation (Table 1). The collapse of the [3Fe–4S] cluster and a corresponding increase in the [4Fe–4S] cluster indicated that the unstable [3Fe–4S] cluster of [3Fe–4S]-TtuA spontaneously transformed to stable [4Fe–4S] ([4Fe–4S]-TtuA) in ten minutes.

### 2.2. Enzymatic Activity of Oxidized TtuA was Recovered by Reconstitution of [4Fe–4S]-TtuA

We analyzed the enzymatic activity of oxidized [4Fe–4S]-TtuA under strictly controlled anaerobic conditions. Because [3Fe–4S] transforms to [4Fe–4S] cluster within one hour (Figure 1d), limiting the reaction time is important. We optimized the reaction conditions, including TtuA and TtuB concentrations and temperature, to monitor the enzymatic activity in a relatively short reaction time. 

**Figure 2:**
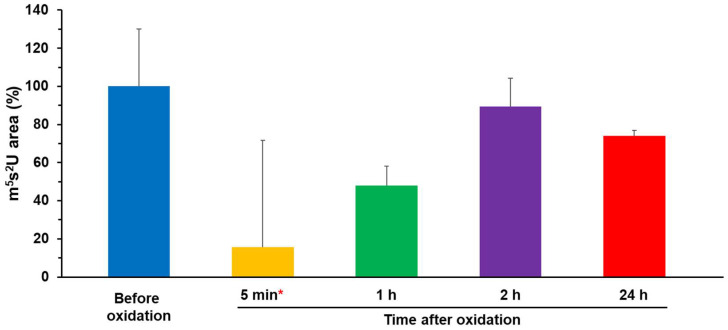
tRNA-thiolation activity assay of [3Fe–4S]-TtuA and [4Fe–4S]-TtuA. Activity assay of [3Fe–4S]-TtuA and [4Fe–4S]-TtuA in the absence of K_3_[Fe(CN)_6_]. The tRNA-thiolation activity of TtuA before oxidation was normalized to 100%. All data are presented with standard deviation values (N = 3, red asterisk (*): one time was measured from a different lot and scaling by the activity of [Before oxidation]). The quantification result of this assay is shown in Table 2.

We measured the synthesis of m^5^s^2^U using oxidized [4Fe–4S]-TtuA in the presence and absence of K_3_[Fe(CN)_6_]. While m^5^s^2^U was detectable using [4Fe–4S]-TtuA, nearly no m^5^s^2^U was synthesized at 5 min after oxidation, indicating that [3Fe–4S]-TtuA had no enzymatic activity. In contrast, the amount of m^5^s^2^U synthesis increased with time (1, 2, and 24 h after oxidation), irrespective of the fact that excess K_3_[Fe(CN)_6_] was removed from the solution (Figure 2 and Figure S6). When TtuA activity was normalized to 100% before oxidation, it was restored to 30%–50% in 1 h, >70% in 2 h, and 70%–80% in 24 h (Table 2). Combining these observations with EPR results, according to which [3Fe–4S] was transformed to [4Fe–4S] cluster with time (Figure 1d, Table 1), it is apparent that TtuA activity increased as the amount of [4Fe–4S] cluster bound to TtuA increased. Furthermore, TtuA activity did not recover to 100% with or without excess K_3_[Fe(CN)_6_]. This finding indicates that some [3Fe–4S] clusters in TtuA degraded into free Fe and S, resulting in the reconstitution of [4Fe–4S]-TtuA and the formation of apo-TtuA. 

### 2.3. The Unique Fe in [4Fe–4S]-TtuA Is Required for its Binding to the Sulfur Donor

**Figure 3:**
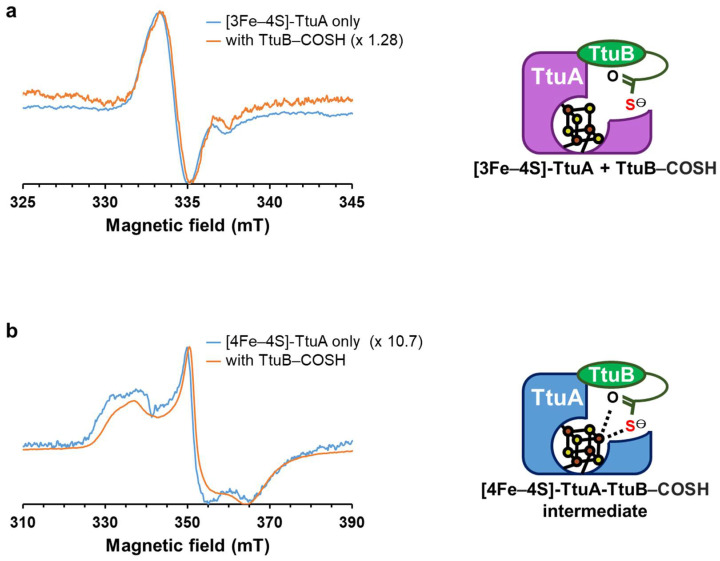
Spectroscopic characterization of [3Fe–4S]-TtuA and [4Fe–4S] with or without the sulfur donor TtuB (TtuB–COSH). (**a**) [3Fe–4S]-TtuA in the absence (blue) and presence (orange) of TtuB–COSH. (**b**) [4Fe–4S]-TtuA in the absence (blue) and presence (orange) of TtuB–COSH [24]. The EPR spectra are normalized for comparison and the raw data are shown in Figure S8.

By monitoring a Fe–S cluster in TtuA under strictly anaerobic conditions in a time-course experiment, we clarified that TtuA activity only requires a [4Fe–4S] cluster. Superposition of the structures of [4Fe–4S]-TtuA-TtuB and apo-TtuA-TtuB showed that the Fe–S cluster binding does not affect the interaction of TtuA and TtuB, except for the C-terminus of the sulfur donor TtuB–COSH (Figure S7). Therefore, to comprehend the function of the unique Fe in [4Fe–4S]-TtuA and evaluate the reason behind the inactivation of [3Fe–4S]-TtuA, we analyzed the interaction between [3Fe–4S]-TtuA (freshly oxidized [4Fe–4S]-TtuA) and TtuB–COSH (sulfur donor) using EPR spectroscopy. First, we obtained the EPR spectra of the [3Fe–4S] cluster with *g*~2.01 from [3Fe–4S]-TtuA, confirming that the unique Fe is absent in the Fe–S cluster of TtuA. Then, we measured the EPR spectra of [3Fe–4S]-TtuA with TtuB–COSH, [4Fe–4S]-TtuA, and [4Fe–4S]-TtuA with TtuB–COSH. We found no significant change in the spectra between [3Fe–4S]-TtuA and [3Fe–4S]-TtuA with TtuB–COSH (Figure 3a and Figure S8a). In contrast, the addition of TtuB–COSH to [4Fe–4S]-TtuA altered the shape and intensity of the spectra, exhibiting the change in the redox potential when the C-terminus hydrosulfide of TtuB–COSH binds to [4Fe–4S]-TtuA (Figure 3b and Figure S8b) [24]. The structure of [4Fe–4S]-TtuA-TtuB complex, in which the C-terminus of TtuB directly coordinates with the unique Fe of [4Fe–4S]-TtuA [24] and the EPR results, indicated that [3Fe–4S]-TtuA cannot bind to the C-terminus of sulfur donor TtuB–COSH. Therefore, the unique Fe of [4Fe–4S]-TtuA is necessary to coordinate with the sulfur donor TtuB–COSH for the transfer of sulfur atoms to the substrate tRNA.

## 3. Discussion

### 3.1. Formation of a Functional Fe–S Cluster Bound to TtuA

Enzymes possess a wide variety of Fe–S clusters. Identification of the Fe–S cluster type is a primary step in understanding the catalytic mechanism of an enzyme. To clarify whether TtuA requires [4Fe–4S] or both [4Fe–4S] and [3Fe–4S] clusters, we performed time-resolved EPR spectroscopy under strictly anaerobic conditions. The results showed that [3Fe–4S]-TtuA, which lacks the unique Fe, was unstable and spontaneously transformed to [4Fe–4S]-TtuA within one hour using [3Fe–4S] cluster from [3Fe–4S]-TtuA as the Fe donor (Figure 1). Because apo-TtuA is an inactive form [25,26] and the Fe source comes from [3Fe–4S] cluster degradation, the maximum recovered activity of [4Fe–4S]-TtuA can be expected to be 75%, and the remaining 25% [3Fe–4S]-TtuA transformed to apo-TtuA. Our experiment showed that the activity of TtuA was restored to approximately 75%, implying that the 25% [3Fe–4S]-TtuA was degraded to further provide a Fe source. Consequently, the most unstable [3Fe–4S]-TtuA (75%) was transformed into [4Fe–4S]-TtuA in 2 h, and the transformation of [3Fe–4S]-TtuA to [4Fe–4S]-TtuA nearly disappeared after 2 h. 

**Figure 4:**
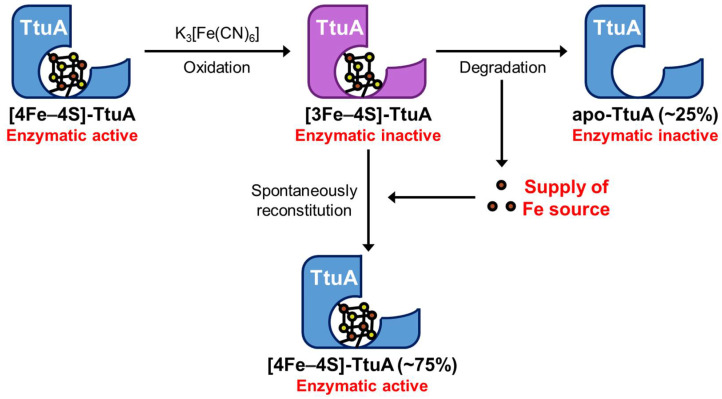
Schematic diagram of the proposed changes of the Fe–S cluster bound to TtuA.

Furthermore, we demonstrated that [3Fe–4S]-TtuA is inactive and cannot bind to the C-terminus of sulfur donor TtuB–COSH (Figure 3 and Figure S8). The enzymatic activity of [3Fe–4S]-TtuA (oxidized [4Fe–4S]-TtuA) recovered with an increase in [4Fe–4S]-TtuA, which was reconstituted spontaneously from [3Fe–4S]-TtuA (Figure 2 and Figure S6). Enzymatic activity did not completely recover because the reaction mixture contained apo-TtuA, which was generated by the degradation of [3Fe–4S] cluster (Figure 4). Considered together, we conclude that only [4Fe–4S]-TtuA has enzymatic activity, and the unique Fe is essential as a binding site for the sulfur donor TtuB–COSH for transferring sulfur to tRNA.

One [3Fe–4S]-TtuA molecule provides three free Fe^3+^ to inactive [3Fe–4S]-TtuA molecules to form active [4Fe–4S]-TtuA. The parentheses indicate the maximum ratio of apo-TtuA and [4Fe–4S]-TtuA.

### 3.2. The Active form of Fe–S Clusters in tRNA-Thiolation Enzymes

Our findings revealed that only [4Fe–4S]-TtuA is the enzymatically active form. Interestingly, some studies reported that TtuA/Ncs6 family members (TtuA/TtcA/Ncs6) bind to [4Fe–4S] cluster [24,25,26,27,28], whereas others have indicated that the active form of Ncs6 and Thil (TtuA homolog) is [3Fe–4S] cluster [29]. Notably, the mechanism of sulfur transfer in TtuA and Ncs6 is believed to be similar [34] because their sulfur donors are homologous proteins, although TtuA and Ncs6 catalyze thiolation at different positions of tRNA [35,36]. To analyze whether the [4Fe–4S] cluster is a feature unique to TtuA or if it is common among tRNA-thiolation enzymes, we compared amino acids around the active site of the TtuA/Ncs6 family members, MnmA, and ThiI, which contain a PP-loop in the PPase domain and a Fe–S cluster per molecule. Although MnmA and ThiI do not belong to the TtuA/Ncs6 family (Table S2), according to recent reports, MnmA from Escherichia coli (*Eco*MnmA) [30,31] may possess [4Fe–4S] cluster in the active site and ThiI from Methanococcus maripaludis (*Mma*ThiI) requires [3Fe–4S] cluster [29].

Previous studies have verified that key residues S55, D59, C130, C133, K137, D161, and C222 in TtuA are involved in tRNA-thiolation [24,37] (Figure S9). The essential residues S55 and D59 in the PP-loop motif are responsible for ATP binding and hydrolysis. D161 is also a critical residue for ATP binding/hydrolysis, though it is 5-Å away from ATP. K137 is the catalytic residue involved in the desulfurization of TtuB–COSH and adenylation of tRNA [24,26]. C130, C133, and C222 are coordinated with the [4Fe–4S] clusters [25]. The sequence alignment of these key residues in tRNA-thiolation enzymes, TtuA/Ncs6 family members, MnmA, and ThiI showed that they are highly conserved in all these proteins, with a few exceptions, such as G56 (D190 in the pyrophosphate binding PP-loop of *Mma*ThiI) and C130 (D99/D101 coordinated to [4Fe–4S] cluster in *Eco*MnmA/*Bsu*MnmA) (Figure S9). Although one of the three cysteines is replaced by aspartic acid in some MnmA, MnmA binds [4Fe–4S] cluster through the aspartic acid instead of cysteine, as seen in ferredoxin, Fnr, IscA, and BchB [10,31,38]. 

We compared the positions of key residues S55, D59, C130, C133, K137, D161, and C222 in tRNA-thiolation enzymes by superposing crystal structures of *Tth*TtuA with those of *Mma*Ncs6, *Eco*MnmA, and models of *Eco*TtcA and *Mma*ThiI (Fe–S cluster type) which was predicted using AlphaFold [39]. The superimpositions showed that all key residues, excluding D161 and C222 of TtuA, are located at similar positions (Figure S10). Conservation of key residues and structural similarity of the active site reveals that [4Fe–4S] cluster binding of TtuA is likely to be shared by members of the TtuA/Ncs6 family, MnmA, and *Mma*ThiI. Our results are consistent with the previous studies, which have reported that TtcA and MnmA are [4Fe–4S]-binding enzymes [40].

### 3.3. The Change of Fe–S Clusters under Strict Regulation of Oxidation–Reduction 

Fe–S clusters are present in diverse forms in the Fe–S proteins. Fe–S clusters are sensitive to oxidation and thus decay readily. Their instability causes incorrect structural determination of Fe–S clusters even with the smallest amount of oxygen contaminant during the experiments. Our findings demonstrated that the Fe–S cluster bound to TtuA is sensitive to oxidation–reduction levels. The [3Fe–4S] clusters quickly and spontaneously transformed into [4Fe–4S] clusters, even under strictly anaerobic conditions. Unexpectedly, in TtuA, the [3Fe–4S] cluster is more unstable than the [4Fe–4S] cluster, which is more oxygen-sensitive and has a unique Fe. Hence, misidentification of the Fe–S clusters may happen without correlation analysis between the structure of Fe–S clusters and the enzymatic activity. Therefore, it is necessary to strictly maintain anaerobic conditions and perform time-course tracking to comprehensively understand the reaction mechanism. 

Many proteins that employ the Fe–S cluster are overlooked as apo-type proteins because Fe–S clusters are easily degraded due to their oxygen sensitivity. Although bioinformatics approaches have recently been used to predict the structure and binding site of Fe–S clusters, experiments are required to confirm these results [41]. The current study demonstrated that [3Fe–4S]-TtuA is inactive and spontaneously transforms into an active form of [4Fe–4S]-TtuA. A time-course analysis of the structure of Fe–S clusters under controlled anaerobic conditions minimizes the risk of incorrectly predicting the reaction mechanism and leads to an accurate understanding of the catalytic mechanism of enzymes with Fe–S clusters.

## 4. Materials and Methods

### 4.1. Expression of TtuA

TtuA, from *Thermus thermophilus* (HB27 strain), was expressed as a C-terminal His6-tagged protein in *E. coli* (B834 DE3 strain) using the pET26 vector expression system (Novagen) [25]. We cultured recombinant *E. coli* in 3 L of lysogeny broth (Miller) containing 25 µg/mL kanamycin at 37 °C and 150 rpm until the absorption at 600 nm (OD600) reached 0.6. Then we induced overexpression of TtuA with 1 mM isopropyl β-D-1-thiogalactopyranoside (IPTG) after cold shock and cultured the cells at 25 °C and 150 rpm for 16 h. The cells were collected by centrifugation at 5000× *g* for 30 min and stored at −30 °C.

### 4.2. Purification of TtuA

Lysis of recombinant *E. coli* and purification of TtuA were performed under strictly anaerobic conditions (Vinyl Anaerobic Chamber, COY) with 5% hydrogen and 95% nitrogen as described [25]. The collected cells were lysed by sonication on ice for 45 min in the purification buffer (50 mM HEPES-KOH [pH 7.6], 200 mM ammonium sulfate, 50 mM ammonium acetate, 5 mM magnesium chloride, 10% (*v*/*v*) glycerol, and 7 mM 2-mercaptoethanol) containing 0.1% Triton X-100. Then the cells were heat treated at 70 °C for 20 min, and the precipitates were removed by centrifugation at 7000× *g* for 1 h with a 0.22-µm filter (Millipore). The supernatant was loaded onto a Ni-affinity chromatography column (1 mL His-Trap HP; GE Healthcare) equilibrated with the purification buffer. Non-specifically bound proteins were removed using the wash buffer (purification buffer containing 50 mM imidazole). The target proteins were eluted with a gradient of 50–500 mM imidazole in the purification buffer and further purified on an SEC column (HiLoad 16/60 Superdex 200, GE Healthcare) equilibrated with the purification buffer. Purity was checked using 15% SDS-PAGE and luminescence images were analyzed with the Amersham Imager 680 (GE Healthcare).

### 4.3. Reconstitution of [4Fe–4S]-TtuA and [3Fe–4S]-TtuA

All steps to reconstitute [4Fe–4S]-TtuA and [3Fe–4S]-TtuA were performed under strictly anaerobic conditions. The concentration of TtuA was measured with the Nanodrop DU 1000 U (Thermo Fisher, Waltham, MA, USA), and UV was used to check for the presence of Fe–S clusters in purified TtuA.

For reconstituting [4Fe–4S]-TtuA, we incubated TtuA with 5 mM dithiothreitol (DTT) for 10 min at room temperature (RT). Then, 9-fold molar excess of ferric chloride (FeCl_3_) was added, and the mixture was incubated for 10 min at RT. Subsequently, 9-fold molar excess Na_2_S was added, and the mixture was incubated for 3 h at RT. The iron sulfide precipitate was removed by centrifugation at 7000× *g* for 10 min with a 0.22-µm filter. Reconstituted TtuA was concentrated using the Amicon Ultra Centrifugal Filter (30-kDa cutoff; Millipore). Excess FeCl_3_ and Na_2_S were removed using the Sephadex PD-10 desalting column (GE Healthcare) equilibrated with the purification buffer.

For reconstituting [3Fe–4S]-TtuA, 6-fold molar excess K_3_[Fe(CN)_6_] was added to [4Fe–4S]-TtuA and incubated at RT for 10 min. The precipitates were removed using centrifugation at 7000× *g* for 30 s and a 0.22-µm filter. K_3_[Fe(CN)_6_] and free Fe were removed with a Sephadex PD-10 desalting column equilibrated with the purification buffer. All treatments were performed under anaerobic conditions (oxygen concentration was less than 1 ppm). Oxidized TtuA was immediately used for EPR or activity assay.

To quantify [4Fe–4S] and [3Fe–4S] in each sample, we double integrated the EPR spectra with the peak areas of [4Fe–4S] and [3Fe–4S]. For estimating the maximum amount of [3Fe–4S] regardless of the quick structural change of [3Fe–4S] to [4Fe–4S], we plotted the signal intensity of [3Fe–4S] versus time after oxidation, and then performed linear approximation by using samples up to 2 h after oxidation (Figure 1e). We calculated the R^2^ value with Microsoft Excel 2019 and found that the result (0.9276) was reliable.

### 4.4. Expression and Purification of TtuB–COSH

TtuB from *T. thermophilus* (HB27 strain) was expressed as a C-terminal intein-tagged protein in *E. coli* (B834 DE3 strain) with the pTYB1 vector (New England BioLabs) as described [25]. We cultured recombinant *E. coli* in 3 L of lysogeny broth containing 100 μg/mL ampicillin at 37 °C and 150 rpm until OD600 reached 0.6. Then we induced overexpression of recombinant TtuB with 1 mM IPTG after cold shock and cultured the cells at 25 °C and 150 rpm for 16 h. The cells were collected by centrifugation at 5000× *g* for 30 min and stored at −30 °C.

We sonicated the collected cells for 30 min in the purification buffer (20 mM Tris-HCl [pH 8.5 at 25 °C] and 500 mM NaCl) with 0.1% Triton X-100, 0.5 mg/mL lysozyme (Sigma), and 0.1 mg/mL DNase I (Sigma). The precipitates were removed by centrifugation at 40,000× *g* for 30 min with a 0.22-µm filter. The supernatant was loaded onto a chitin resin (New England BioLabs) equilibrated with the purification buffer. Non-specifically bound proteins were removed using 20 column volumes of the purification buffer. To cleave the intein tag from the TtuB–intein fusion protein, 1 column volume of the purification buffer containing 50 mM ammonium sulfide ((NH_4_)_2_S) was added to the chitin resin and incubated at RT for 20 h.

TtuB was eluted in a cold room (7 °C) with the purification buffer and then concentrated using the Amicon Ultra Centrifugal Filter (3-kDa cutoff). Excess (NH_4_)_2_S was removed with the Sephadex PD-10 desalting column equilibrated with the storage buffer (14 mM Tris-HCl [pH 8.5] at 25 °C, 350 mM NaCl, and 30% (*v*/*v*) glycerol). The fractions containing TtuB were collected, concentrated using the Amicon Ultra Centrifugal Filter (3-kDa cutoff), and stored at −80 °C. We confirmed the purity of TtuB by 15% (*v*/*v*) SDS-PAGE.

To confirm the presence of sulfur at the C-terminus of TtuB, we removed salts from TtuB solution on ice with the C4 ZipTip (Millipore) and performed matrix-assisted laser desorption/ionization-time-of-flight mass spectrometry (MALDI-TOF MS) using UltraflexIII (Bruker) with sinapinic acid as the matrix [25]. The concentration of TtuB was determined by the Bradford method [42], using Protein Assay Dye Reagent Concentrate (Bio-rad) because TtuB from *T. thermophilus* does not contain tyrosine and tryptophan.

### 4.5. EPR Spectroscopy of the Fe–S Cluster Bound to TtuA

To analyze the structure of the Fe–S cluster bound to TtuA in time-course, we prepared EPR samples under strictly anaerobic conditions. Immediately after [3Fe–4S]-TtuA was prepared, we divided fresh 0.5 mM [3Fe–4S]-TtuA into 14 samples (200 µL/sample). For analyzing [3Fe–4S]-TtuA, DT was not added to the sample 5 min after preparation of [3Fe–4S]-TtuA (sample 1). To analyze [4Fe–4S]-TtuA, we added 5-fold molar excess DT to the sample 5 min after preparation of [3Fe–4S]-TtuA (sample 2) and incubated the sample at 25 °C for 10 min. To prevent degradation of the Fe–S clusters, samples 1 and 2 were aliquoted into quartz EPR tubes (Agri) and frozen simultaneously with liquid nitrogen in the anaerobic chamber. Similarly, we froze samples 10 min (samples 3 and 4), 20 min (samples 5 and 6), 30 min (samples 7 and 8), 1 h (samples 9 and 10), 2 h (samples 11 and 12), 12 h (samples 13 and 14), and 24 h (samples 15 and 16) after preparation of [3Fe–4S]-TtuA (Figure S11). Moreover, 200 µL of 0.5 mM [4Fe–4S]-TtuA was frozen as control to analyze the amount of [4Fe–4S]-TtuA before oxidation (sample 0). We conducted EPR experiments twice: one was the measurement in 0, 5, 10, 20, 30, 60, 120 min, and 24 h, and the other was a long time (0, 5, 30, 60 min, 12 h, and 24 h).

Because [4Fe–4S]^2+^ and Fe^2+^ are EPR-silent, it is necessary to change [4Fe–4S]^2+^ of [4Fe–4S]-TtuA to [4Fe–4S]^1+^ by adding reductant DT to obtain the spectra for [4Fe–4S]-TtuA [25]. Because free Fe^3+^ is reduced to Fe^2+^ under DT-reduced conditions, we could not detect free Fe^3+^ in reduced [4Fe–4S] samples. 

To analyze the interaction between [3Fe–4S]-TtuA and TtuB–COSH, we prepared EPR samples using a slightly modified version of our protocol [24]. Fresh [3Fe–4S]-TtuA was mixed with TtuB to a final concentration of 0.5 mM and incubated at 25 °C for 5 min under strictly anaerobic conditions. The sample mixture was incubated with a 5-fold molar excess DT at 25 °C for 10 min, aliquoted into quartz EPR tubes, and then frozen. The EPR samples were stored in liquid nitrogen and transported to the Center for Experimental Science and Analysis, Saga University, or Institute for Molecular Science, Okazaki. Continuous wave (CW) X-band EPR spectra were measured in the CW mode at ~9.59 GHz using the ELEXSYS E580 spectrometer (Bruker) equipped with the ESR 910 continuous helium flow cryostat (Oxford Instruments) and E500 spectrometer (Bruker) equipped with the ESR 900 continuous helium flow cryostat (Oxford Instruments). The experimental parameters were 12 K for both [4Fe–4S]^1+^ and free Fe^3+^, and 40 K for [3Fe–4S]^1+^; 1 mW microwave power; 100 kHz field modulation; and 10 G modulation amplitude.

### 4.6. Activity Assay for [3Fe–4S]-TtuA and [4Fe–4S]-TtuA 

The formation of m^5^s^2^U on substrate tRNA was conducted under strictly anaerobic conditions as described [24]. The standard assay was performed in 30 µL of reaction buffer (50 mM HEPES-KOH [pH 7.6], 100 mM KCl, 10 mM MgCl_2_, and 0.1 mM DTT) containing 2.5 µM TtuA, 15 µM brewer’s yeast total tRNA (Sigma), 15 µM TtuB, and 5 mM ATP at 60 °C for 10 min. Because [3Fe–4S] in TtuA quickly changes to [4Fe–4S], it was necessary to initiate tRNA-thiolation immediately after removing K_3_[Fe(CN)_6_] from TtuA solution and limit reaction time. In our previous study, we incubated 4-fold molar excess of TtuB (20 µM) with 5 µM of TtuA at 60 °C for 30 min [24]. In this study, we obtained a final concentration of 2.5 µM for TtuA and analyzed the time-course of enzymatic activity (Figure S12). To detect enzyme activity even at low concentrations of TtuA and short reaction times, we added 6-fold molar excess of TtuB (15 µM). 

tRNA-thiolation was stopped with 120 µL stop buffer (75 µL Isogen (Nippon Gene) and 45 µL deionized water) and then frozen at −30 °C until tRNA extraction. tRNA was extracted with phenol:chloroform (5:1 [pH 4.5]; Thermo Fisher), precipitated with ethanol, and digested with digestion buffer (100 mM HEPES-NaOH [pH 7.5]) containing 6.2 mU/µL nuclease P1 (Yamasa) and 5.0 mU/µL bacterial alkaline phosphatase (Takara Bio). The concentration of digested tRNA was measured with the Nanodrop 2000 (Thermo Fisher). The sample solution was loaded onto an Inertsil ODS-3 column (2.1 mm × 150 mm × 3 μm; GL Science) equilibrated with HPLC buffer (0.1% formic acid and 2% acetonitrile). The target nucleoside (m^5^s^2^U) was eluted with a gradient of 2%–40% acetonitrile. The amount of m^5^s^2^U was detected with UV light at 280 nm using the Extrema HPLC system (Jasco Inc., Easton, MD, USA).

## Figures and Tables

**Table 1:** Spectroscopic quantification of the Fe–S cluster bound to TtuA.

Time after Oxidation	[3Fe–4S]	[4Fe–4S]
Before oxidation	―	100%
Immediately after oxidation (0 min)	(100%)	―
5 min ^†^	99% ± 7%	12% ± 1%
10 min	76%	16%
20 min	76%	19%
30 min ^†^	70% ± 8%	20% ± 4%
1 h ^†^	50% ± 13%	30% ± 8%
2 h *	28%	33%
12 h	7%	77%
24 h ^†^	9% ± 6%	77% ± 14%

EPR measurements were performed twice under conditions marked with daggers (†) and one time without the dagger. The standard deviation was calculated by N = 2. The parentheses indicate the estimated amount from the calibration curve (Figure 1e). The asterisk (*) indicates a weak signal with a relatively large experiment error.

**Table 2:** Biochemical quantification of the enzymatic activity of [3Fe–4S]-TtuA and [4Fe–4S]-TtuA.

Time after Oxidation	Enzymatic Activity without K_3_[Fe(CN)_6_]
Before oxidation	100% ± 30%
5 min *	16% ± 56%
1 h	48% ± 10%
2 h	89% ± 15%
24 h	74% ± 3%

All data were measured three times and shown with standard deviation. Asterisk (*): one time was measured from a different lot and scaling by the activity of [Before oxidation].

## Data Availability

Not applicable.

## Supplementary Materials

The following supporting information can be downloaded at: https://www.mdpi.com/article/10.3390/ijms24010833/s1, Figure S1: Sequence alignment of tRNA-thiolation enzymes; Figure S2: Scheme of thiolated tRNA biosynthesis catalyzed by tRNA-thiolation enzymes; Figure S3: Redox states of TtuA and their detectability with EPR; Figure S4: EPR spectroscopic characterization of TtuA with excess DT; Figure S5: [3Fe–4S]^1+^ EPR spectra of [3Fe–4S]-TtuA with excess K_3_[Fe(CN)_6_]; Figure S6: Activity assay of [3Fe–4S]-TtuA and [4Fe–4S]-TtuA in the presence of K_3_[Fe(CN)_6_]; Figure S7: Structural alignment of the [4Fe–4S]-TtuA-TtuB complex and the apo–TtuA-TtuB complex; Figure S8: EPR spectra comparation of TtuA with/without TtuB–COSH; Figure S9: Sequence alignment of key residues of TtuA for tRNA-thiolation; Figure S10: Structural alignment of tRNA-thiolation enzymes; Figure S11: Anaerobic preparation of EPR and assay samples in time-course; Figure S12: Schematic showing the time scale of sample preparation for the activity assay; Table S1: Characterization of reported tRNA-thiolation enzymes with Fe–S clusters; Table S2: Sequence identity of tRNA-thiolation enzymes. References [43,44,45,46,47] are citied in the Supplementary Materials.
