# Supplementary material for: Quick and Spontaneous Transformation between [3Fe–4S] and [4Fe–4S] Iron–Sulfur Clusters in the tRNA-Thiolation Enzyme TtuA
Source: Int J Mol Sci. 2023 Jan 3;24(1):833. doi: 10.3390/ijms24010833 (PMC9821441; doi:10.3390/ijms24010833)
Supplement: Supplementary file 1 [file ijms-24-00833-s001.zip › ijms-2058748-supplementary.pdf]

# Supplementary Information

# Quick and spontaneous transformation between [3Fe–4S] and [4Fe–4S] iron–sulfur clusters in the tRNA-thiolation enzyme TtuA

Masato Ishizaka[1], Minghao Chen[2], Shun Narai[1], Yoshikazu Tanaka[2,3], Toyoyuki Ose[1,2], Masaki Horitani[4,5,*], and Min Yao[1,2,*]

[1]Graduate School of Life Science, Hokkaido University, Kita 8, Nishi 5, Kita-ku, Sapporo, Hokkaido, 060-0810, Japan

[2]Faculty of Advanced Life Science, Hokkaido University, Kita 8, Nishi 5, Kita-ku, Sapporo, Hokkaido, 060-0810, Japan

[3]Graduate School of Life Sciences, Tohoku University, 2-1-1 Katahara, Aoba-ku, Sendai, 980-8577, Japan

[4]Faculty of Agriculture, Department of Applied Biochemistry and Food Science, Saga University, 1 Hon-jo-machi, Saga, 840-8502, Japan

[5]The United Graduate School of Agricultural Science, Kagoshima University, 1-21-24 Korimoto, Kagoshima, 890-0065, Japan

[*]Correspondence:

yao@castor.sci.hokudai.ac.jp

horitani@cc.saga-u.ac.jp

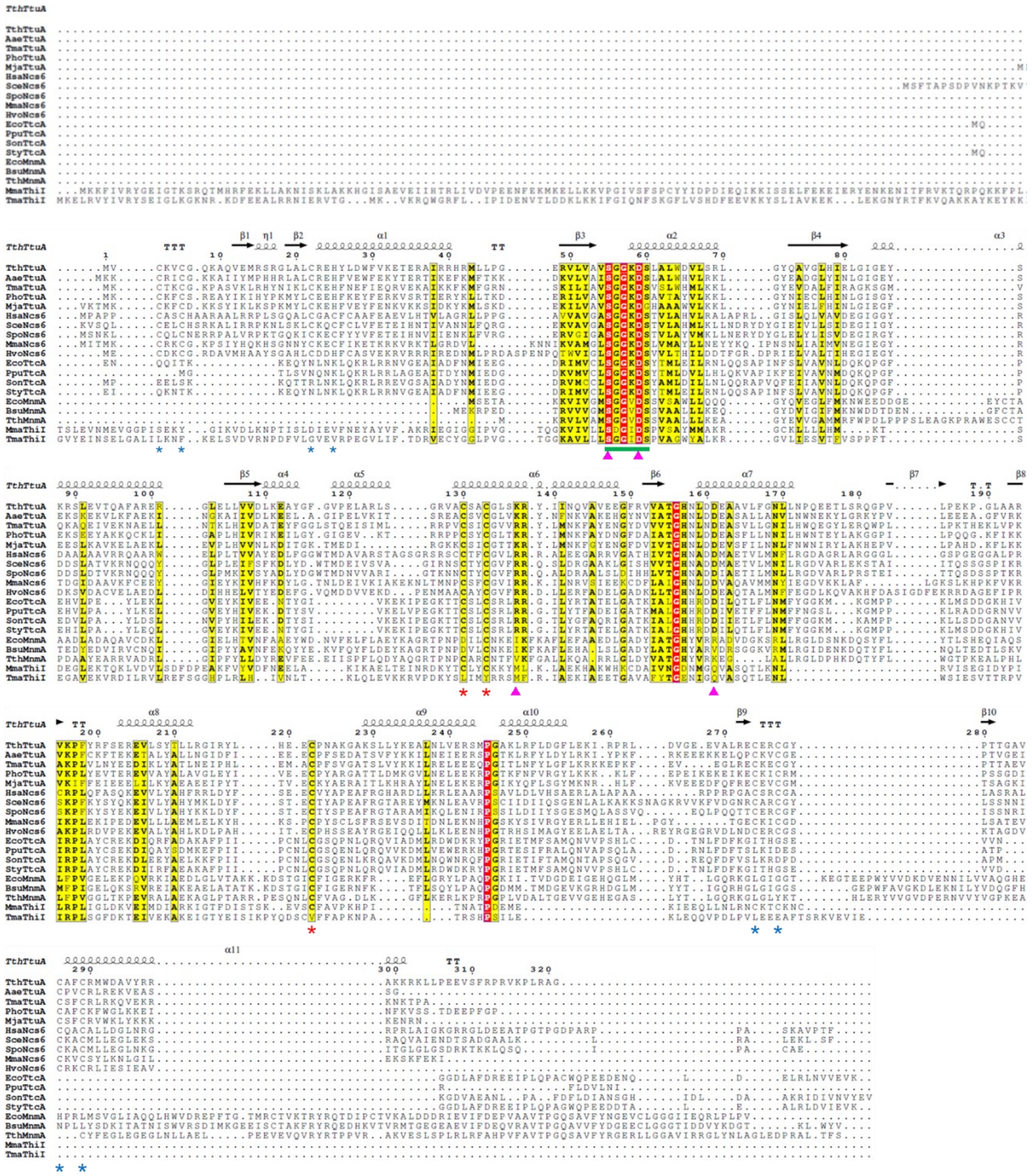

**Supplementary Figure S1.** Sequence alignment of tRNA-thiolation enzymes.

The secondary structures of *Tth*TtuA (PDB ID: 5B4F) are shown at the top. Completely conserved residues are highlighted in red and highly conserved residues are highlighted in yellow, respectively. Key residues in the ATP-binding motif (PP-loop, a green line); the three cysteine residues bound to the Fe–S cluster (red asterisks); the cysteine or histidine residues involved in the tRNA-binding motif (Zn finger, blue asterisks); and key

residues of *Tth*TtuA identified in previous studies [1,2] (magenta triangles). The alignment was performed using Clustal Omega [3] and ESPrint 3.0 [4] was used for illustration. Note that V345 of *Tma*ThiI aligns with C222 of *Tth*TtuA because their sequence identity is only 13.9%; however, C344 is the catalytic residue of *Tma*ThiI [5]. The abbreviations of protein names are the same as that in Table S2.

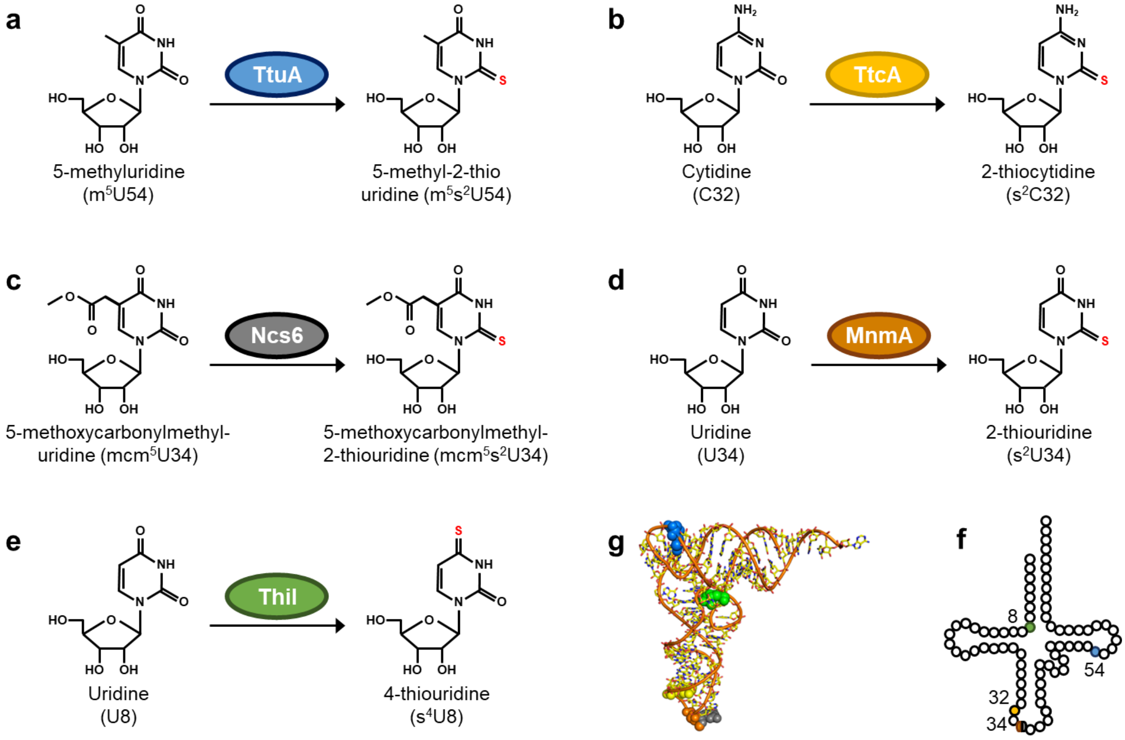


**Supplementary Figure S2.** Scheme of thiolated tRNA biosynthesis catalyzed by tRNA-thiolation enzymes.

TtuA [6,7], TtcA [8], and Ncs6 [9,10] (TtuA/Ncs6 family members) catalyze the formation of 5-methyl-2-thiouridine (**a**), 2-thiocytidine (**b**), and 5-methoxycarbonylmethyl-2-thiouridine (**c**), respectively. Although MnmA [11] and ThiI [12] do not belong to the TtuA/Ncs6 family, Fe–S clusters are essential for bacterial MnmA and *Methanococcus* ThiI to catalyze the formation of 2-thiouridine (**d**) and 4-thiouridine (**e**), respectively. For clarity, the modifications sites of tRNA-thiolation in 3D (PDB code: 1EHZ) (**g**) and 2D (**f**) structures are shown. Note that some thiolations cannot occur simultaneously.

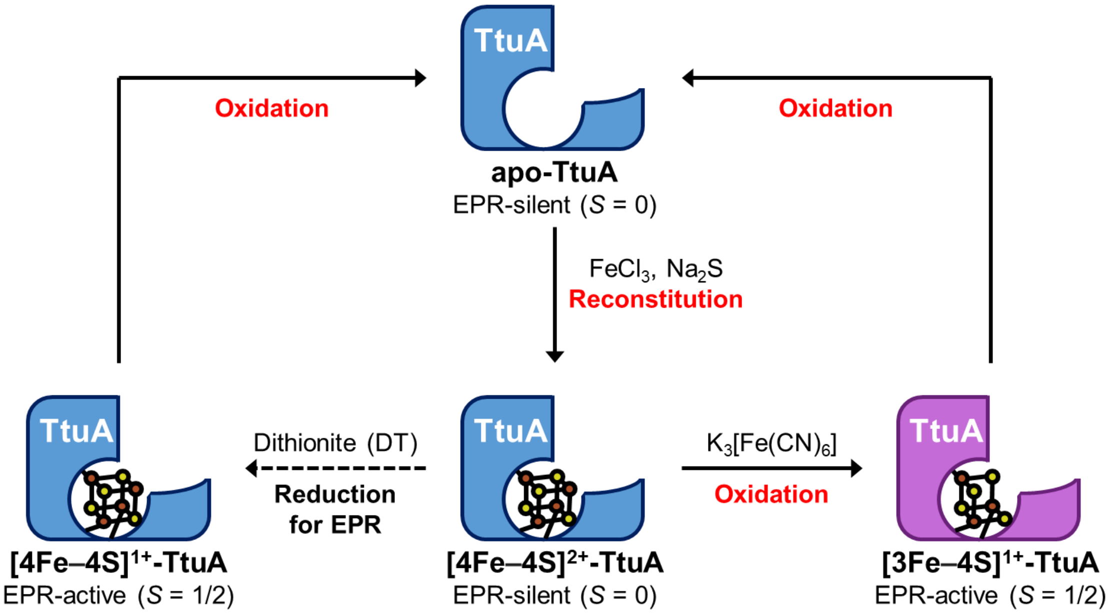


**Supplementary Figure S3.** Redox states of TtuA and their detectability with EPR. Reconstructed TtuA is +2 charged with spin quantum number $S$ = 0 and EPR-silent. Reduction using dithionite (DT) makes [4Fe–4S]-TtuA EPR-active. Oxidation with $K_3[Fe(CN)_6]$ enables EPR detection of [3Fe–4S]-TtuA.

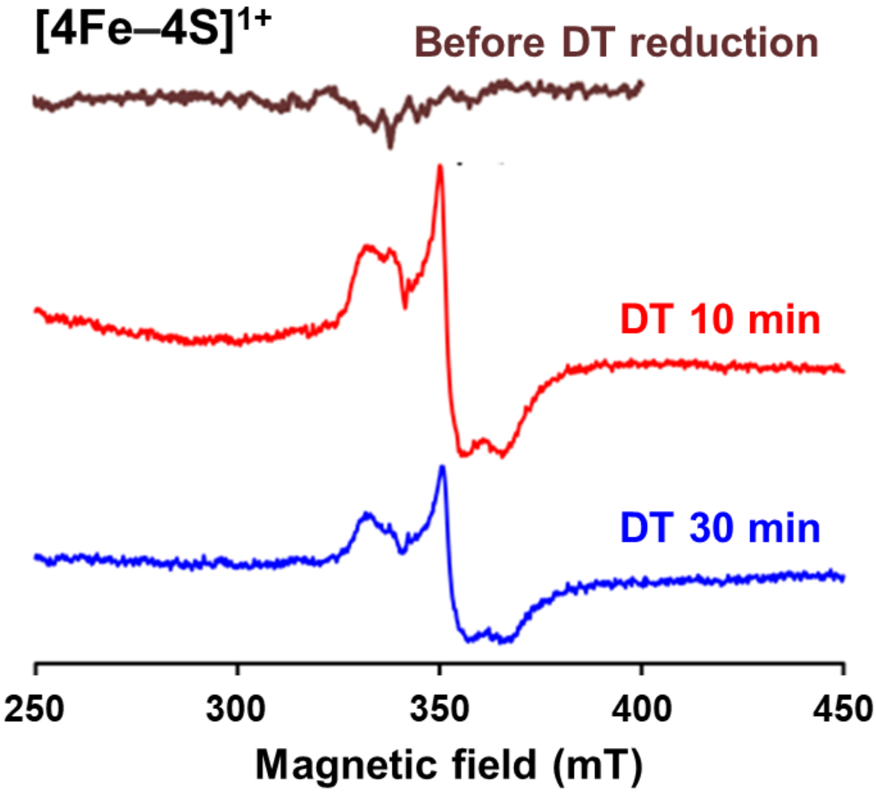


**Supplementary Figure S4.** EPR spectroscopic characterization of TtuA with excess DT. EPR spectra of [4Fe–4S]-TtuA before DT reduction (brown) and reduced by DT and incubated for 10 min (red) and 30 min (blue). $[4Fe–4S]^{1+}$ was detected in the presence of DT at 12 K.

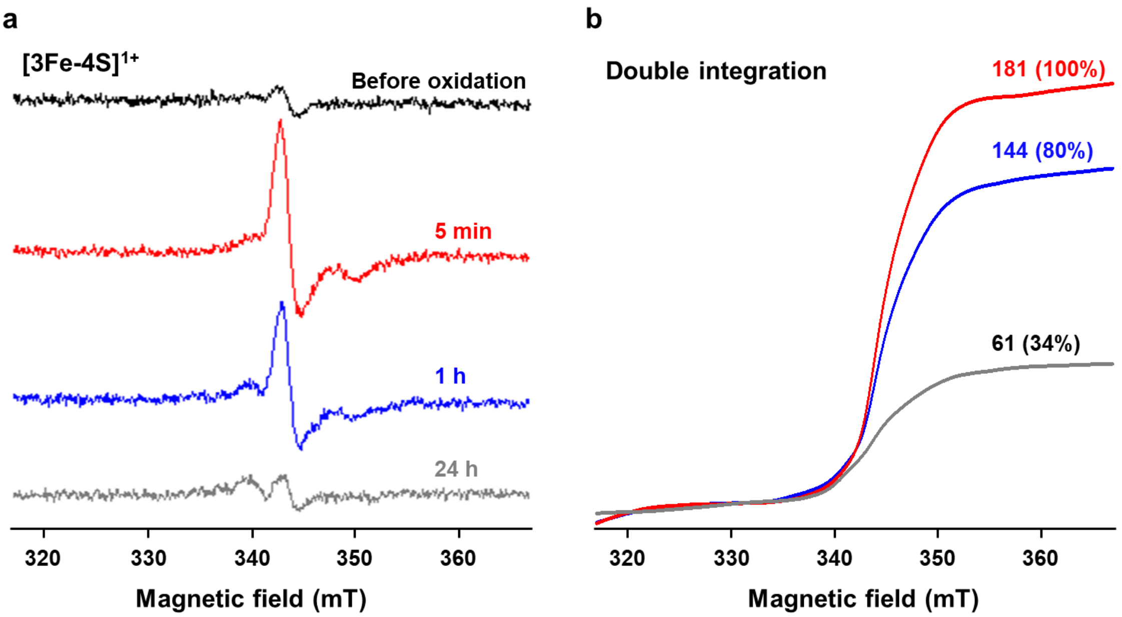


**Supplementary Figure S5.** $[3Fe–4S]^{1+}$ EPR spectra of [3Fe–4S]-TtuA with excess $K_3[Fe(CN)_6]$.

(**a**) [4Fe–4S]-TtuA was oxidized to [3Fe–4S]-TtuA with excess $K_3[Fe(CN)_6]$ (no desalination) and incubated for 5 min (red), 1 h (blue), and 24 h (gray). The sample before oxidization ([4Fe–4S]-TtuA) was used as a reference (black). $[3Fe–4S]^{1+}$ was detected in the absence of DT at 40 K. (**b**) Double integration of (a). The EPR signal intensity ratio is shown in parentheses, where the EPR signal intensity at 5 min after oxidation is 100%.

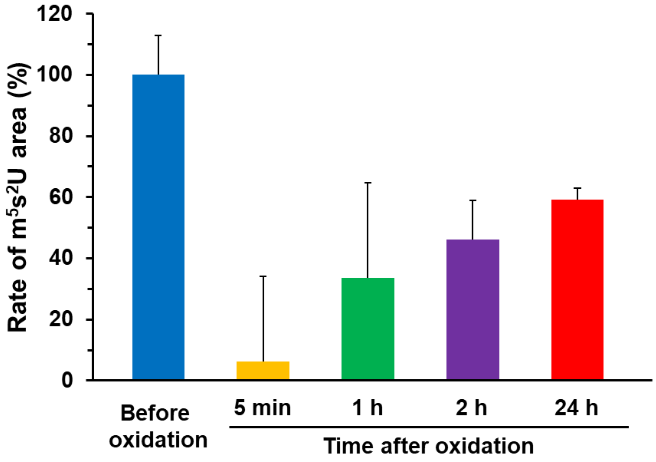


| Time after oxidation | Enzymatic activity |
| --- | --- |
| Before oxidation | 100%±13% |
| 5 min | 6%±28% |
| 1 h | 34%±31% |
| 2 h | 46%±13% |
| 24 h | 59%±4% |

**Supplementary Figure S6.** Activity assay of [3Fe–4S]-TtuA and [4Fe–4S]-TtuA in the presence of $K_3[Fe(CN)_6]$.

The tRNA thiolation activity of TtuA before oxidation ([4Fe–4S]-TtuA) was normalized to 100%. All data are presented in the right panel with standard deviation values ($N = 3$).

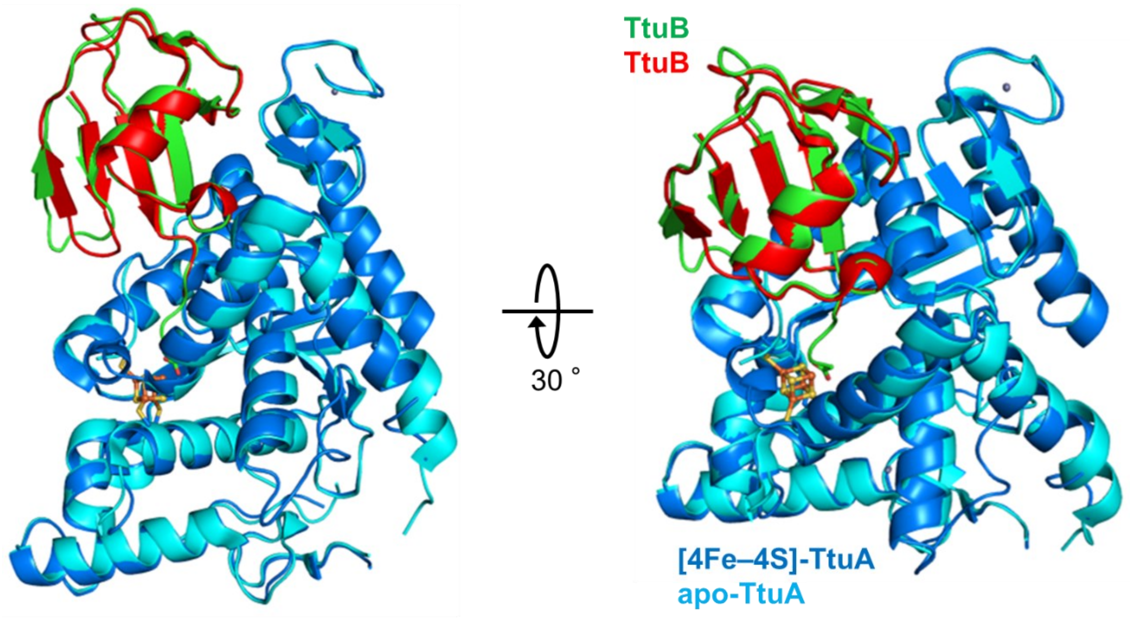


**Supplementary Figure S7.** Structural alignment of the [4Fe–4S]-TtuA-TtuB complex and the apo–TtuA-TtuB complex.

TtuA and TtuB of the [4Fe–4S]-TtuA-TtuB complex (PDB ID: 5ZTB) are colored in blue and green, respectively [1]. TtuA and TtuB of the apo-TtuA-TtuB complex (PDB ID: 5GHA) are colored in cyan and red, respectively [7]. The Cα root-mean-square deviation (RMSD) value is 0.483 Å, which is calculated by “align” command of PyMOL ver1.7. The two C-terminal residues of TtuB are disordered when [4Fe–4S] is absent.

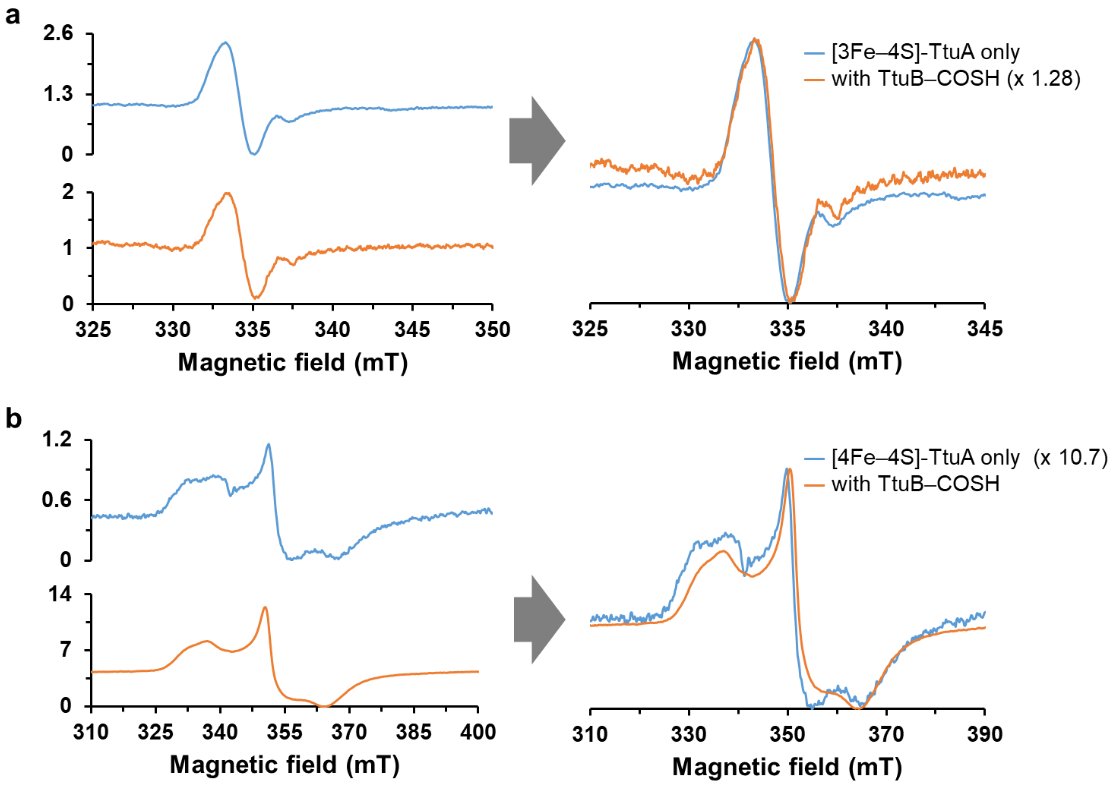


**Supplementary Figure S8.** EPR spectra comparation of TtuA with/without TtuB–COSH. (**a**) Raw data (left) and normalized data (right) of [3Fe–4S]-TtuA in the absence (blue) and presence (orange) of the sulfur donor TtuB–COSH. (**b**) Raw data (left) and normalized data (right) of [4Fe–4S]-TtuA in the absence (blue) and presence (orange) of the sulfur donor TtuB–COSH [1]. These normalized data are the same as in Figure 3.

| Enzymes | Organisms | Key residues | | | | | | | | | | | | | | | |
|---|---|---|---|---|---|---|---|---|---|---|---|---|---|---|---|---|---|
| TtuA | *T. thermophilus* | S55 | G56 | G57 | K58 | D59 | S60 | … | C130* | … | C133* | … | K137 | … | D161 | … | C222* |
| TtuA | *A. aeolicus* | S56 | G57 | G58 | K59 | D60 | S61 | … | C130* | … | C133* | … | K137 | … | D161 | … | C222* |
| TtuA | *T. maritima* | S55 | G56 | G57 | K58 | D59 | S60 | … | C131* | … | C134* | … | R138 | … | D162 | … | C224* |
| TtuA | *P. horikoshii* | S55 | G56 | G57 | K58 | D59 | S60 | … | C128* | … | C131* | … | K135 | … | D159 | … | C220* |
| TtuA | *M. jannaschii* | S59 | G60 | G61 | K62 | D63 | G64 | … | C130* | … | C133* | … | K137 | … | D161 | … | C222* |
| TtcA | *E. coli* | S47 | G48 | G49 | K50 | D51 | S62 | … | C122* | … | C125* | … | R129 | … | D153 | … | C213* |
| TtcA | *P. putida* | S40 | G41 | G42 | K43 | D44 | S45 | … | C115* | … | C118* | … | R122 | … | D146 | … | C206* |
| TtcA | *S. oneidensis* | S45 | S46 | G47 | K48 | D49 | S50 | … | C120* | … | C123* | … | R127 | … | D151 | … | C211* |
| TtcA | *S. typhimurium* | S47 | G48 | G49 | K50 | D51 | S52 | … | C122* | … | C125* | … | R129 | … | D153 | … | C213* |
| Ncs6 (Ctu1) | *H. sapiens* | S59 | G60 | G61 | K62 | D63 | S64 | … | C144* | … | C147* | … | R151 | … | D175 | … | C237* |
| Ncs6 (Ncs6p) | *S. cerevisiae* | S75 | G76 | G77 | K78 | D79 | S80 | … | C157* | … | C160* | … | R164 | … | D188 | … | C250* |
| Ncs6 (Ncs6p) | *S. pombe* | S59 | G60 | G61 | K62 | D63 | S64 | … | C142* | … | C145* | … | R149 | … | D173 | … | C235* |
| Ncs6 (NcsA) | *M. maripaludis* | S57 | G58 | G59 | K60 | D61 | S62 | … | C142* | … | C145* | … | R149 | … | D173 | … | C233* |
| Ncs6 (NcsA) | *H. volcanii* | S61 | G62 | G63 | K64 | D65 | S66 | … | C143* | … | C146* | … | R150 | … | D174 | … | C242* |
| MnmA | *E. coli* | S13 | G14 | G15 | V16 | D17 | S18 | … | D99* | … | C102* | … | I106 | … | R132 | … | C199* |
| MnmA | *B. subtilis* | S15 | G16 | G17 | V18 | D19 | S20 | … | D101* | … | C104* | … | I108 | … | V133 | … | C200* |
| MnmA | *T. thermophilus* | S9 | G10 | G11 | V12 | D13 | S14 | … | C105* | … | C108* | … | V112 | … | K137 | … | C200* |
| ThiI | *M. maripaludis* | S189 | D190 | G191 | I192 | D193 | S194 | … | C265* | … | C268* | … | M272 | … | Q296 | … | C348* |
| ThiI | *T. maritima* | S182 | G183 | G184 | I185 | D186 | S187 | … | L260 | … | Y263 | … | M267 | … | Q291 | … | C344 |

**Supplementary Figure S9.** Sequence alignment of key residues of TtuA for tRNA-thiolation.

Key residues in *Tth*TtuA have been identified in other studies [1,2]. The residues are strongly conserved between the TtuA/Ncs6 family members (TtuA, TtcA, and Ncs6). Residues with asterisks are coordinated to Fe–S clusters. The abbreviations of source organisms are the same as that in Table S2. Note that only the PP-loop of *Mma*ThiI is not completely conserved (G190D) and *Eco*MnmA/*Bsu*MnmA uses D99/D101 instead of cysteines to bind [4Fe–4S]. V345 of *Tma*ThiI aligns with C222 of *Tth*TtuA in Figure S1, but C344 in this figure because C344 is one of the catalytic residues of *Tma*ThiI [5].

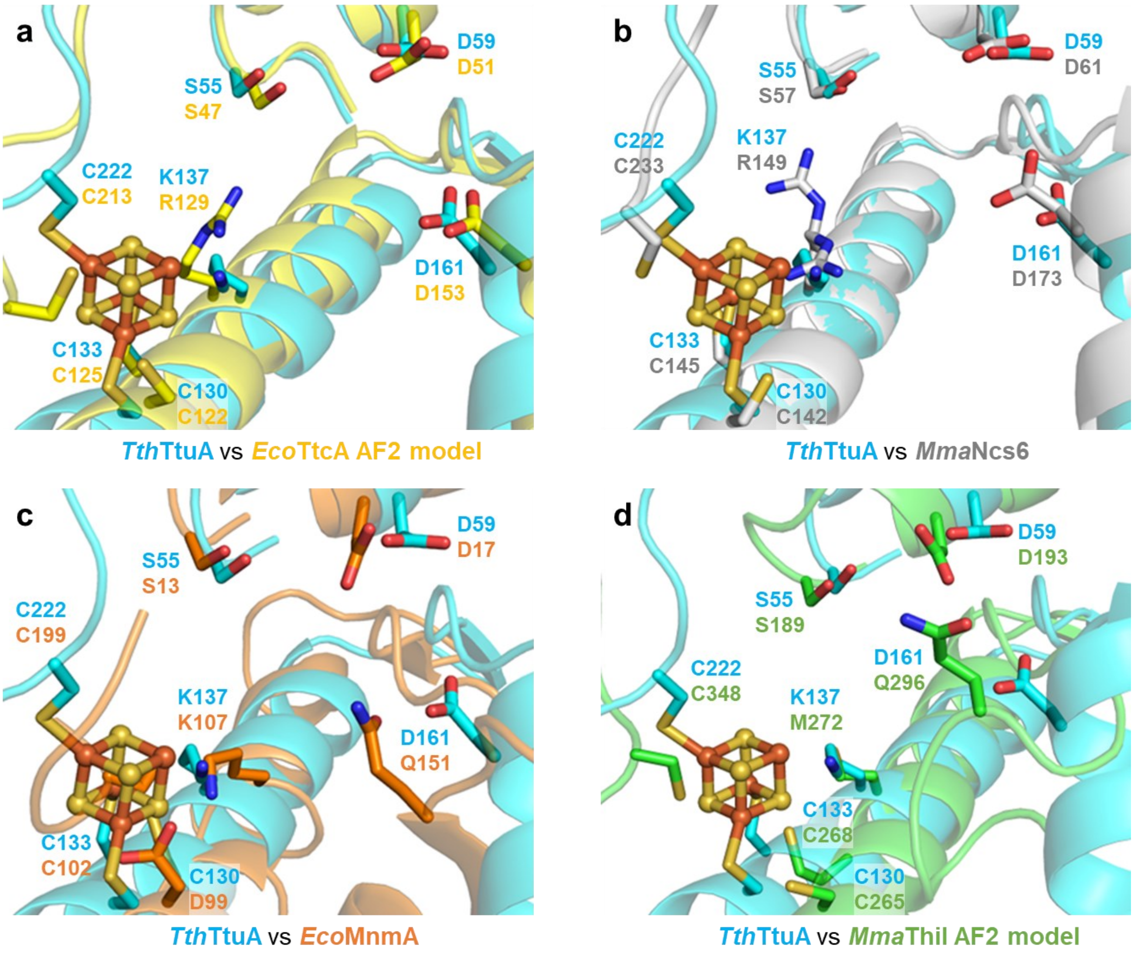


**Supplementary Figure S10.** Structural alignment of tRNA-thiolation enzymes.

Superposition of *Tth*TtuA (PDB ID: 5B4F) with the (**a**) *Eco*TtcA predicted model, (**b**) *Mma*Ncs6 (PDB ID: 6SCY), (**c**) *Eco*MnmA (PDB ID: 2DEU), and (**d**) *Mma*ThiI (Fe–S cluster type) predicted model. The models in (**a**) and (**d**) were predicted using the local version of AlphaFold ver2.0 (AF2) [13] with default parameters. We used the models with the highest reliability based on the predicted local-distance difference test (pLDDT) score between the five predicted structures for each enzyme. TtuA family members TtcA and Ncs6 were superimposed with TtuA using the "align" command, whereas MnmA and PPase domain of *Mma*ThiI (G182-H354) were superimposed with TtuA using the "super" command of PyMOL ver1.7.

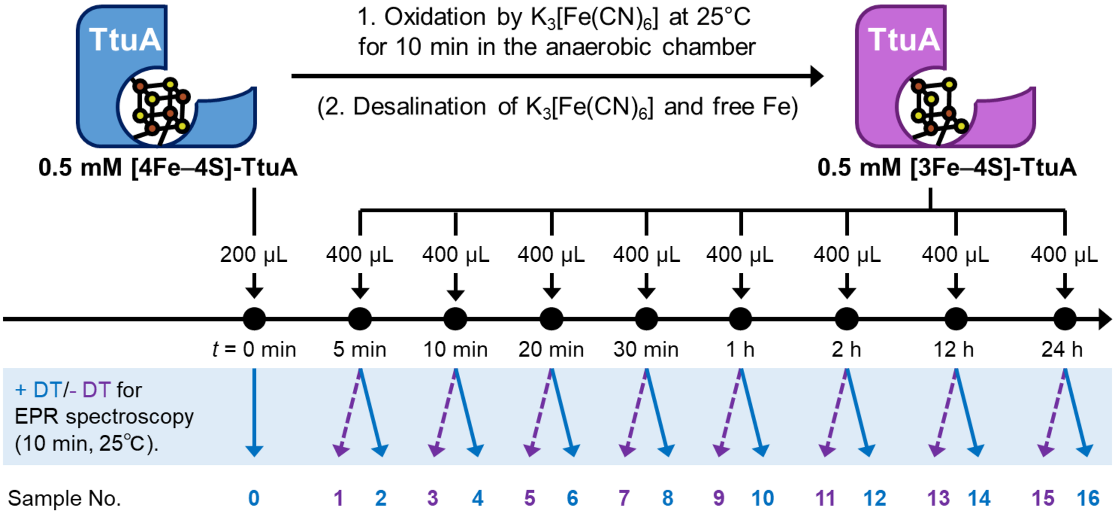


**Supplementary Figure S11.** Anaerobic preparation of EPR and assay samples in time-course.

Even-numbered EPR samples contain DT for evaluating [4Fe–4S]-TtuA (blue solid arrows). Odd-numbered EPR samples do not contain DT for evaluating [3Fe–4S]-TtuA (purple dashed arrows). For EPR spectroscopy, desalination is an optional step. For the activity assay, all samples were incubated at 60°C (not 25°C) for 10 min immediately after the preparation of oxidized TtuA ([3Fe–4S]-TtuA). The colors of EPR samples (sample volume is 200 µL) are the same as in Figure 1d.

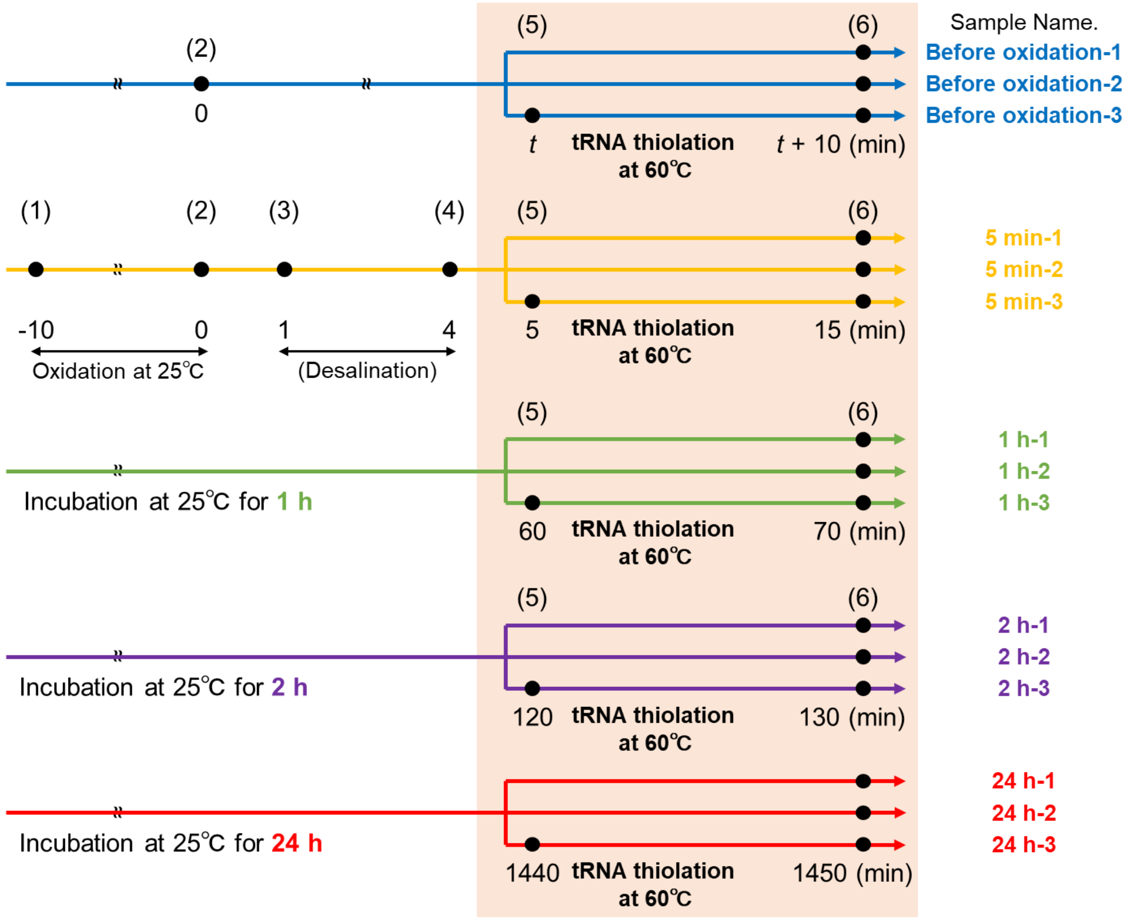


**Supplementary Figure S12.** Schematic showing the time scale of sample preparation for the activity assay.

(**1**) Addition of $K_3[Fe(CN)_6]$ to [4Fe–4S]-TtuA for preparing oxidized TtuA ([3Fe–4S]-TtuA). Oxidation time was 10 min at 25°C. (**2**) Centrifugation to remove precipitation. (**3**) Sample loading to desalination column to remove $K_3[Fe(CN)_6]$ and free Fe. (**4**) Mixture of desalted sample. (**5**) Addition of TtuA to premix reaction solution and incubation at 60°C for 10 min. (**6**) Anaerobic termination of tRNA-thiolation by adding 120 µL of the stop buffer. The color code of each sample is the same as in Figure 2.

**Supplementary Table S1.** Characterization of reported tRNA-thiolation enzymes with Fe–S clusters.

| Enzyme | Cofactor | Species | Modification | Position |
|---|---|---|---|---|
| TtuA | [4Fe–4S] | Thermophilic bacterium and thermophilic archaeon | 5-methyl-2-thiouridine ($m^5s^2U$) | 54 (T-loop) |
| TtcA | [4Fe–4S] | Bacterium | 2-thiocytidine ($s^2C$) | 32 (Anticodon) |
| Ncs6 | [4Fe–4S] or [3Fe–4S] | Archaeon and eukaryote | 5-methoxycarbonylmethyl-2-thiouridine ($mcm^5s^2U34$) | 34 (Anticodon) |
| MnmA | [4Fe–4S] | Bacterium | 2-thiouridine ($s^2U$) | 34 (Anticodon) |
| ThiI | [4Fe–4S] or [3Fe–4S] | Bacterium and archaeon | 4-thiouridine ($s^4U$) | 8 (D-arm) |

$m^5s^2U$: 5-methyl-2-thiouridine

$s^2C$: 2-thiocytidine

$mcm^5s^2U$: 5-methoxycarbonylmethyl-2-thiouridine

$s^2U$: 2-thiouridine

$s^4U$: 4-thiouridine

TtuA: 2-thiouridine synthetase

TtcA: 2-thiocytidine synthetase

Ncs6: 2-thiouridine synthetase

MnmA: 2-thiouridine synthetase

ThiI: 4-thiouridine synthetase

**Supplementary Table S2.** Sequence identity of tRNA-thiolation enzymes.

| | *Aae* TtuA | *Tma* TtuA | *Pho* TtuA | *Mja* TtuA | *Eco* TtcA | *Ppu* TtcA | *Son* TtcA | *Sty* TtcA | *Hsa* Ncs6 | *Sce* Ncs6 | *Spo* Ncs6 | *Mma* Ncs6 | *Hvo* Ncs6 | *Eco* MnmA | *Bsu* MnmA | *Tth* MnmA | *Mma* ThiI | *Tma* ThiI |
|---|---|---|---|---|---|---|---|---|---|---|---|---|---|---|---|---|---|---|
| *Tth* TtuA | 45.5 (74.0) | 37.0 (71.6) | 39.6 (72.9) | 33.5 (67.7) | 17.4 (52.6) | 18.5 (48.0) | 17.4 (50.0) | 16.6 (52.6) | 27.1 (52.9) | 23.6 (56.1) | 22.6 (59.8) | 23.4 (57.8) | 23.8 (54.7) | 13.2 (44.6) | 11.3 (40.2) | 15.6 (40.9) | 12.8 (47.3) | 13.9 (46.2) |
| *Aae* TtuA | | 47.3 (76.1) | 44.3 (75.5) | 39.1 (71.7) | 19.2 (52.9) | 20.4 (54.9) | 17.2 (50.6) | 18.3 (52.3) | 22.5 (49.3) | 23.4 (51.3) | 22.6 (54.8) | 26.9 (60.9) | 29.0 (63.3 | 12.1 (41.2) | 11.3 (42.1) | 13.1 (39.1) | 12.8 (44.7) | 14.8 (45.7) |
| *Tma* TtuA | | | 41.1 (76.1) | 41.3 (74.8) | 18.0 (50.2) | 17.7 (51.5) | 16.8 (51.1) | 18.1 (51.5) | 20.7 (50.3) | 23.4 (53.8) | 22.7 (59.1) | 26.0 (60.2) | 30.3 (59.6) | 10.5 (30.9) | 10.2 (39.1) | 11.8 (40.6) | 14.8 (46.2) | 14.3 (42.5) |
| *Pho* TtuA | | | | 50.6 (80.6 | 17.5 (51.4) | 18.0 (49.8) | 16.7 (51.1) | 18.3 (51.9) | 25.1 (52.3) | 27.3 (55.2) | 25.7 (58.8) | 30.2 (62.2) | 27.5 (59.7) | 12.4 (41.2) | 13.2 (44.0) | 12.5 (40.9) | 12.8 (43.6) | 13.4 (43.8) |
| *Mja TtuA* | | | | | 18.9 (50.9) | 19.9 (51.3) | 19.2 (51.4) | 18.4 (50.5) | 21.7 (50.6) | 23.4 (55.4) | 24.1 (60.7) | 27.1 (62.5) | 26.7 (61.5) | 12.8 (42.9) | 12.3 (43.2) | 11.7 (42.0) | 15.6 (45.1) | 12.6 (45.9) |
| *Eco* TtcA | | | | | | 59.5 (78.5 | 60.8 (85.7) | 94.5 (99.7) | 17.7 (47.6) | 16.9 (54.7) | 17.7 (52.7) | 19.8 (53.0) | 20.4 (51.4) | 10.8 (41.5) | 13.6 (44.1) | 10.5 (38.1) | 12.5 (47.4) | 13.6 (44.2) |
| *Ppu* TtcA | | | | | | | 63.8 (80.7) | 59.2 (78.8) | 19.9 (49.0) | 17.2 (49.2) | 17.8 (49.9) | 21.1 (53.8) | 21.6 (54.6) | 12.0 (39.7) | 14.0 (39.4) | 14.5 (41.1) | 12.7 (40.9) | 12.5 (42.5) |
| *Son* TtcA | | | | | | | | 61.5 (85.0) | 19.1 (49.0) | 18.7 (52.4) | 18.2 (52.4) | 18.5 (50.5) | 20.9 (51.6) | 11.7 (42.6) | 12.7 (42.6) | 14.0 (44.7) | 12.5 (45.7) | 12.3 (40.8) |
| *Sty* TtcA | | | | | | | | | 18.8 (48.7) | 18.9 (53.9) | 17.7 (51.5) | 20.1 (52.1) | 20.4 (49.0) | 12.7 (45.4) | 13.6 (43.3) | 10.8 (38.8) | 12.5 (44.8) | 15.9 (44.3) |
| *Hsa* Ncs6 | | | | | | | | | | 43.6 (72.2) | 42.9 (74.2) | 26.9 (58.2) | 29.5 (56.8) | 11.8 (44.0) | 13.3 (44.7) | 15.0 (41.0) | 11.6 (39.3) | 11.4 (43.2) |
| *Sce* Ncs6 | | | | | | | | | | | 58.3 (80.0) | 25.6 (59.2) | 29.3 (57.8) | 11.6 (44.2) | 12.6 (42.4) | 12.8 (42.4) | 11.3 (47.7) | 12.9 (50.1) |
| *Spo* Ncs6 | | | | | | | | | | | | 28.0 (62.8) | 28.3 (59.1) | 10.2 (44.7) | 8.3 (44.8) | 10.3 (44.1) | 13.0 (44.4) | 12.2 (50.1) |
| *Mma* Ncs6 | | | | | | | | | | | | | 33.7 (68.1) | 11.7 (39.6) | 10.7 (42.9) | 12.7 (44.1) | 14.3 (43.9) | 12.1 (45.5) |
| *Hvo* Ncs6 | | | | | | | | | | | | | | 13.3 (46.2) | 13.5 (44.3) | 12.8 (42.7) | 12.0 (42.1) | 11.0 (44.4) |
| *Eco* MnmA | | | | | | | | | | | | | | | 54.3 (79.5) | 37.7 (65.4) | 10.2 (43.0) | 12.4 (44.7) |
| *Bsu* MnmA | | | | | | | | | | | | | | | | 36.6 (66.5) | 11.3 (45.5) | 12.3 (46.9) |
| *Tth* MnmA | | | | | | | | | | | | | | | | | 9.3 (43.8) | 12.2 (42.5) |
| *Mma* ThiI | | | | | | | | | | | | | | | | | | 31.9 (66.1) |

The upper value indicates identity (%) and the lower value indicates similarity (%) of the amino acid sequence evaluated using ClastalW [14]. Identity values ≥40%; ≥30% but <40%; and ≥20% but <30% are highlighted in red, yellow, and green, respectively. TtuA from *Thermus thermophilus* HB27 (*Tth*TtuA), *Aquifex aeolicus* VF5 (*Aae*TtuA), *Thermotoga maritima* MSB8 (*Tma*TtuA), *Pyrococcus horikoshii* OT-3 (*Pho*TtuA), and *Methanocaldococcus jannaschii* ATCC 43067 (*Mja*TtuA) [6,7]. TtcA from *Escherichia coli* K12 (*Eco*TtcA), *Pseudomonas putida* W619 (*Ppu*TtcA), *Shewanella oneidensis* MR-1 (*Son*TtcA), and *Salmonella typhimurium* LT2 (*Sty*TtcA) [8]. Ncs6 from *Homo sapiens* (*Hsa*Ncs6), *Saccharomyces cerevisiae* ATCC 204508 (*Sce*Ncs6), *Schizosaccharomyces pombe* 972 strain (*Spo*Ncs6), *Methanococcus maripaludis* S2 (*Mma*Ncs6), and *Haloferax volcanii* ATCC 29605 (*Hvo*Ncs6) [9,10]. MnmA from *E. coli* K12 (*Eco*MnmA), *Bacillus subtilis* 168 (*Bsu*MnmA), and *T. thermophilus* HB27 (*Tth*MnmA) [11]. ThiI from *T. maritima* MSB8 (*Tma*ThiI) and *M. maripaludis* S2 (*Mma*ThiI) [9,12]. All amino acid sequences were obtained from UniProt [15].

## References


1. Chen, M.; Ishizaka, M.; Narai, S.; Horitani, M.; Shigi, N.; Yao, M.; Tanaka, Y. The [4Fe-4S] cluster of sulfurtransferase TtuA desulfurizes TtuB during tRNA modification in Thermus thermophilus. *Commun. Biol.* **2020**, *3*, 168, doi:10.1038/s42003-020-0895-3.
2. Nakagawa, H.; Kuratani, M.; Goto-Ito, S.; Ito, T.; Katsura, K.; Terada, T.; Shirouzu, M.; Sekine, S.; Shigi, N.; Yokoyama, S. Crystallographic and mutational studies on the tRNA thiouridine synthetase TtuA. *Proteins Struct. Funct. Bioinforma.* **2013**, *81*, 1232–1244, doi:10.1002/prot.24273.
3. Chenna, R.; Sugawara, H.; Koike, T.; Lopez, R.; Gibson, T.J.; Higgins, D.G.; Thompson, J.D. Multiple sequence alignment with the Clustal series of programs. *Nucleic Acids Res.* **2003**, *31*, 3497–3500, doi:10.1093/nar/gkg500.
4. Robert, X.; Gouet, P. Deciphering key features in protein structures with the new ENDscript server. *Nucleic Acids Res.* **2014**, *42*, 320–324, doi:10.1093/nar/gku316.
5. Neumann, P.; Lakomek, K.; Naumann, P.-T.; Erwin, W.M.; Lauhon, C.T.; Ficner, R. Crystal structure of a 4-thiouridine synthetase–RNA complex reveals specificity of tRNA U8 modification. *Nucleic Acids Res.* **2014**, *42*, 6673–6685, doi:10.1093/nar/gku249.
6. Shigi, N.; Sakaguchi, Y.; Suzuki, T.; Watanabe, K. Identification of two tRNA thiolation genes required for cell growth at extremely high temperatures. *J. Biol. Chem.* **2006**, *281*, 14296–14306, doi:10.1074/jbc.M511675200.
7. Chen, M.; Asai, S.; Narai, S.; Nambu, S.; Omura, N.; Sakaguchi, Y.; Suzuki, T.; Ikeda-Saito, M.; Watanabe, K.; Yao, M.; et al. Biochemical and structural characterization of oxygen-sensitive 2-thiouridine synthesis catalyzed by an iron-sulfur protein TtuA. *Proc. Natl. Acad. Sci. U.S.A.* **2017**, *114*, 4954–4959, doi:10.1073/pnas.1615585114.
8. Bouvier, D.; Labessan, N.; Clémancey, M.; Latour, J.-M.; Ravanat, J.-L.; Fontecave, M.; Atta, M. TtcA a new tRNA-thioltransferase with an Fe-S cluster. *Nucleic Acids Res.* **2014**, *42*, 7960–7970, doi:10.1093/nar/gku508.
9. Bimai, O.; Arragain, S.; Golinelli-Pimpaneau, B. Structure-based mechanistic insights into catalysis by tRNA thiolation enzymes. *Curr. Opin. Struct. Biol.* **2020**, *65*, 69–78, doi:10.1016/j.sbi.2020.06.002.

10. Dewez, M.; Bauer, F.; Dieu, M.; Raes, M.; Vandenhaute, J.; Hermand, D. The conserved wobble uridine tRNA thiolase Ctu1-Ctu2 is required to maintain genome integrity. *Proc. Natl. Acad. Sci. U.S.A.* **2008**, *105*, 5459–5464, doi:10.1073/pnas.0709404105.
11. Shigi, N.; Horitani, M.; Miyauchi, K.; Suzuki, T.; Kuroki, M. An ancient type of MnmA protein is an iron-sulfur cluster-dependent sulfurtransferase for tRNA anticodons. *RNA* **2020**, *26*, 240–250, doi:10.1261/rna.072066.119.
12. Liu, Y.; Vinyard, D.J.; Reesbeck, M.E.; Suzuki, T.; Manakongtreecheep, K.; Holland, P.L.; Brudvig, G.W.; Söll, D. A [3Fe-4S] cluster is required for tRNA thiolation in archaea and eukaryotes. *Proc. Natl. Acad. Sci. U.S.A.* **2016**, *113*, 12703–12708, doi:10.1073/pnas.1615732113.
13. Jumper, J.; Evans, R.; Pritzel, A.; Green, T.; Figurnov, M.; Ronneberger, O.; Tunyasuvunakool, K.; Bates, R.; Žídek, A.; Potapenko, A.; et al. Highly accurate protein structure prediction with AlphaFold. *Nature* **2021**, *596*, 583–589, doi:10.1038/s41586-021-03819-2.
14. Combet, C.; Blanchet, C.; Geourjon, C.; Deléage, G. NPS@: Network protein sequence analysis. *Trends Biochem. Sci.* **2000**, *25*, 147–150, doi:10.1016/S0968-0004(99)01540-6.
15. Apweiler, R.; Bairoch, A.; Wu, C.H.; Barker, W.C.; Boeckmann, B.; Ferro, S.; Gasteiger, E.; Huang, H.; Lopez, R.; Magrane, M.; et al. UniProt: The universal protein knowledgebase. *Nucleic Acids Res.* **2004**, *32*, 2699, doi:10.1093/nar/gky092.
